# Relationship Among Internet Use, eHealth Literacy, Internet Addiction, and Physical Activity Among Adolescents: Cross-Sectional Study

**DOI:** 10.2196/83936

**Published:** 2026-03-30

**Authors:** Xavier C C Fung, Joyce S C Cheung, Fay F Wang, Benson W M Lau, Shirley P C Ngai

**Affiliations:** 1 Department of Rehabilitation Sciences, Faculty of Health and Social Sciences, The Hong Kong Polytechnic University, Hong Kong Special Administrative Region, China, +852 27664801

**Keywords:** eHealth literacy, internet use, internet addiction, physical activity, adolescents

## Abstract

**Background:**

The internet is highly convenient and has become an indispensable part of daily life. However, its widespread use also has notable disadvantages, such as the risk of internet addiction. Still, increased internet use may enhance eHealth literacy, and online health information seeking may contribute to health promotion. In Hong Kong, internet addiction and low physical activity among adolescents are growing concerns, underscoring the need to address internet use to better support the health and well-being of youth.

**Objective:**

This study aimed to investigate the effects of internet use, eHealth literacy, and internet addiction on adolescents’ physical activity in Hong Kong.

**Methods:**

An online cross-sectional study was conducted in Hong Kong between June 2023 and August 2023. Secondary school students aged 12 to 18 years were recruited. Data were collected using the eHealth Literacy Scale, the International Physical Activity Questionnaire, the Chen Internet Addiction Scale, and a questionnaire assessing demographic characteristics and the use of eHealth technologies. Spearman ρ correlation and mediation analyses were performed to examine the relationships among variables.

**Results:**

A total of 117 participants were included. Participants reported an average internet use of 5.28 (SD 3.50) hours per day, and the mean eHealth literacy score was 31.15 (SD 4.04). Correlation analyses revealed that internet use was positively correlated with internet addiction (*r*=0.33; *P*<.001) but negatively correlated with physical activity (*r*=−0.21; *P*=.02), and internet addiction was negatively correlated with physical activity (*r*=−0.26; *P*=.005). In addition, the mediation analysis demonstrated that both internet use and eHealth literacy had a direct effect on internet addiction (*B*=1.53, *P*<.001 and *B*=−0.91, *P*=.002, respectively). Internet addiction had a direct effect on physical activity (*B*=−43.94, *P*=.02). In contrast, eHealth literacy had no significant direct effect on physical activity and did not mediate the relationship between internet use and physical activity.

**Conclusions:**

The findings highlight the importance of eHealth literacy in reducing internet addiction. However, future research should further examine factors that mediate the relationship between eHealth literacy and physical activity or other health-related behaviors. This study sheds light on the benefits of promoting eHealth literacy among adolescents to prevent internet addiction and offers practical insights for teachers and parents.

## Introduction

The internet has transformed modern life and has become an integral part of our daily activities by providing widespread access to information and connectivity [1]. However, these benefits are accompanied by potential drawbacks, including the risk of addiction. Internet addiction is a growing global concern, and a substantial body of research suggests that excessive internet use can lead to various adverse health outcomes, including distress and impairment in daily functioning [2].

In Hong Kong, the prevalence of internet addiction among adolescents ranged from 17% to 26.8% over the 6-year longitudinal study from the 2009 to 2010 to 2015 to 2016 school years [3]. A review by Chung et al [4] found an increasing prevalence, consistent with the upward trend in internet use among adolescents over the past decade. Moreover, internet-related behaviors, including excessive smartphone use, have intensified since the COVID-19 pandemic disrupted daily routines [5]. Adolescents who spend more time using the internet have an increased risk of adverse outcomes, such as decreased physical activity and poor physical development [2]. Overall, these findings highlight the importance of addressing internet and smartphone use among adolescents to support their health and well-being.

The potential impact of internet addiction on physical activity and cardiovascular health has elicited concern [6-9]. Problematic internet use has been associated with physical inactivity among high school students [10,11]. Physical inactivity and sedentary behavior can lead to adverse health problems, such as obesity and cardiovascular disease [12]. For instance, children who engage in sedentary behaviors, such as screen viewing, were found to be approximately 5.68 times more likely to become overweight [13]. Furthermore, adolescents with low physical activity levels were reported to be 7.69 times more likely to have high blood pressure [14]. These findings indicate the need to identify potential protective factors that might reduce internet addiction and promote physical activity.

Longer time spent using the internet may facilitate health information seeking online [15]. eHealth literacy is defined as the ability to seek, find, understand, and appraise health information from electronic sources and to apply the knowledge gained to address or solve health problems [16]. This concept has become increasingly important because the internet has become a major source of health information, and digital health interventions have become more common [17]. Studies have reported that eHealth literacy is a significant predictor of health-promoting behaviors, including physical activity, nutritional behaviors, life appreciation, social support, stress management, and health responsibility, among adolescents [18,19]. These findings highlight the benefits of promoting eHealth literacy among young individuals. Evidence suggests that a poor health status and unhealthy lifestyle during adolescence are associated with poor health in adulthood [20,21]. Thus, eHealth literacy appears to be a protective factor worthy of further investigation. Furthermore, a study reported a negative association between health literacy and internet addiction [22]. To further explain the potential relationships among internet use, eHealth literacy, internet addiction, and physical activity, social cognitive theory provides a useful theoretical framework [23]. This theory emphasizes the dynamic interactions among personal, environmental, and behavioral factors [23]. Within this framework, internet use may function as an environmental factor by providing access to public information and shaping attitudes and norms related to physical activity. Personal factors may be represented by eHealth literacy, which can influence personal beliefs and foster self-efficacy related to adopting physical activity, and by internet addiction, which may reflect low self-efficacy in regulating online behavior and serve as a maladaptive coping strategy that undermines physical activity. Physical activity, in turn, is the behavioral outcome.

Internet addiction and low physical activity levels among adolescents in Hong Kong have become growing concerns [3,24,25]. For instance, the Student Health Service Annual Health Report for the 2023 to 2024 school year reported that 96.1% of secondary school students did not meet the recommended physical activity levels [26]. Furthermore, the proportion of secondary school students engaging in recreational screen time for 2 or more hours per day increased from 78% in the 2022 to 2023 school year to 80.9% in the 2023 to 2024 school year [26]. Enhancing eHealth literacy can help address internet addiction and insufficient physical activity among adolescents. However, although studies on eHealth literacy have been conducted in Hong Kong, they have mainly focused on adults [27,28]. Thus, little is known about eHealth literacy among Hong Kong adolescents. Furthermore, to the best of our knowledge, no study has examined the relationships among eHealth literacy, internet addiction, and physical activity in this population. This gap in the literature warrants further investigation. Evidence indicates that eHealth can positively affect health behaviors, and this potential should be specifically examined in relation to internet addiction and physical activity.

**Figure 1.**
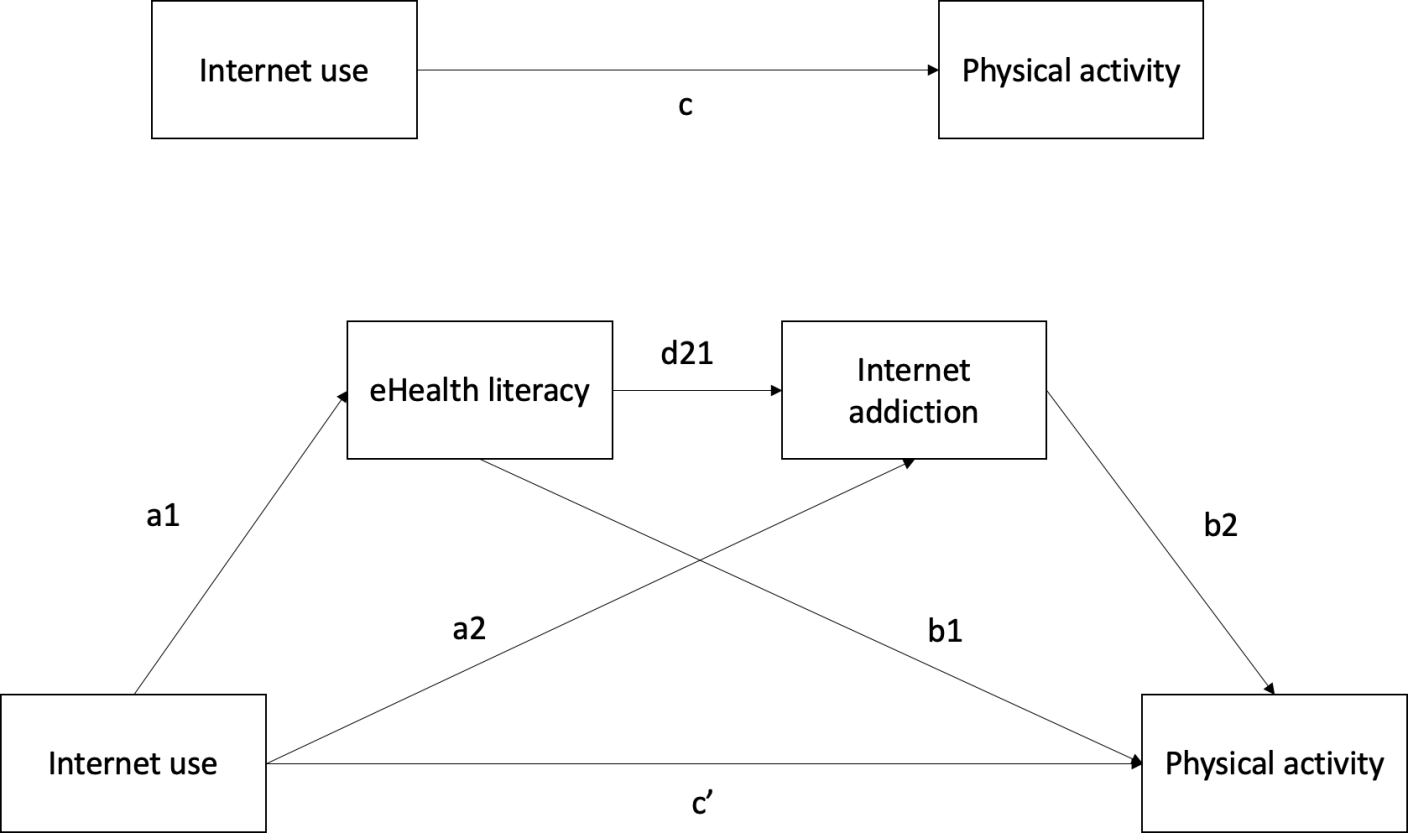
The proposed mediation model for internet use, eHealth literacy, internet addiction, and physical activity.

This study investigated the effects of internet use, eHealth literacy, and internet addiction on physical activity levels among adolescents in Hong Kong. In addition, this study examined the mediating role of eHealth literacy in mitigating the negative effects of internet use and internet addiction on physical activity (Figure 1). Specifically, we hypothesized that (1) internet use is positively associated with eHealth literacy, (2) eHealth literacy is negatively associated with internet addiction, (3) eHealth literacy is positively associated with physical activity, and (4) eHealth literacy and internet addiction mediate the relationship between internet use and physical activity.

## Methods

### Participants

A total of 7 secondary school students who participated in a research internship program at The Hong Kong Polytechnic University assisted with participant recruitment for this study after receiving approval from their respective schools. As the participating secondary schools did not have their own ethics review bodies, ethics approval for the study was obtained from the study institution. Participants were recruited through convenience sampling from the secondary schools attended by the student interns. After the study details were explained to students and their parents, students were invited to complete a set of questionnaires administered through Google Forms. Upon selecting “agree” on the Google Form, participants were directed to the questionnaire section. To minimize duplicate responses, participants were instructed to complete the questionnaire only once using their Google account. Data were collected between June 2023 and August 2023, which coincided with the summer school holiday period in Hong Kong. As students generally have more free time to use digital devices during the holiday, this period was selected for data collection.

The eligibility criteria for participation were as follows: (1) an age of 12 to 18 years, (2) status as a full-time secondary school student in Hong Kong, (3) ability to understand traditional Chinese, and (4) ability to use electronic devices to access the online survey. Individuals who self-reported neurological illnesses (eg, stroke and autism), functional disabilities (eg, blindness), or any form of psychosis or intellectual disability that could hinder completion of the online survey were excluded from participation.

A total of 123 responses were received. Of these, 5 respondents did not provide informed consent, and one did not meet the inclusion criteria. Therefore, 117 responses were included in the analysis. For sample size estimation, a confidence level of 95% was applied, with the margin of error set at 10% because of the relatively short data collection period and the estimated proportion was set to 0.5. On the basis of these parameters, the estimated required sample size was 96.

### Outcome Measures

#### Demographic Information and eHealth Technology Use

Demographic information was collected, including participants’ age, sex, grade, height, and weight. Furthermore, a set of questions was used to evaluate participants’ use of technology and health-related applications, including (1) the amount of time spent on the internet per day and (2) whether technologies were used for health monitoring. The measured domains and the validity and localization testing of the instruments used are summarized in Table 1.

**Table 1.** Summary of instruments used in the test: domains measured and reference on validity testing or translation.

Instruments	Domains measured	Validity test and translation studies
eHealth Literacy Scale	Consumer’s integrative knowledge, comfort, and perceived skills at searching, and evaluation and application of electronic information to address health-related problems	Koo et al [29]
International Physical Activity Questionnaire	Monitor activity habits over the previous 7 days, with focus on leisure, domestic, work, and transport	Macfarlane et al [30]
Chen Internet Addiction Scale	Compulsive use, withdrawal, tolerance, problems in interpersonal relationships, as well as health and time management	Chen et al [31]

#### eHealth Literacy Scale

The eHealth Literacy Scale (eHEALS) is a questionnaire designed to evaluate individuals’ eHealth literacy. It includes items that measure self-perceived competence and confidence in finding, evaluating, and applying health information obtained from electronic sources [16]. This scale comprises 8 items, which are rated on a 5-point Likert scale. This study used the Chinese version of the eHEALS (C-eHEALS) [29]. Koo et al [29] examined the psychometric properties of the C-eHEALS. Additionally, the C-eHEALS was used in a study of adolescents in Hong Kong and demonstrated excellent internal consistency (Cronbach α=0.95) [32]. In this study, the C-eHEALS similarly exhibited high internal consistency (Cronbach α=0.92).

#### International Physical Activity Questionnaire

The Chinese version of the International Physical Activity Questionnaire (IPAQ)–Short Form (IPAQ-C) was used in this study [30]. This self-reported questionnaire assesses the amount of physical activity individuals engaged in during the past week [30]. An example item is as follows: “During the last seven days, on how many days did you participate in vigorous physical activities?” [30]. Responses are converted into metabolic equivalent of task (MET) values based on the duration and intensity of physical activity (MET=3.3 for walking, 4 for moderate physical activity, and 8 for vigorous physical activity) [30]. For example, if an individual walks for 60 minutes per day over 7 days, their MET score is calculated by multiplying 3.3 by 60 minutes and 7 days, yielding a total of 1386 METs. A higher MET score indicates a higher level of physical activity. The IPAQ-C demonstrated satisfactory psychometric properties and was validated among adolescents in Hong Kong [30].

#### Chen Internet Addiction Scale

The Chen Internet Addiction Scale (CIAS) is a self-reported instrument consisting of 26 items designed to assess various symptoms and problems related to internet use [31]. The scale covers 4 main dimensions: compulsive use, withdrawal, tolerance, and problems related to interpersonal relationships, health, and time management. The total CIAS score ranges from 26 to 104. Established cutoff points for screening (57/58) and diagnosis (63/64) are available to classify the severity of internet addiction. In addition, the total score can be used to assess the tendency toward internet addiction, with higher scores indicating greater severity [33]. In this study, only the total CIAS score was used for analysis. The Cronbach α for the CIAS was 0.95 in a previous study conducted in Hong Kong [34] and 0.95 in this study.

### Data Analysis

Data are presented as mean (SD), unless otherwise indicated. After examining the distributional assumption, the normality assumption was found to be violated. Therefore, Spearman ρ correlation was used to examine the relationships between internet addiction, physical activity, eHealth literacy, and internet use among adolescents. A mediation model was then used to examine the role of eHealth literacy as a mediator in the relationships between internet use, internet addiction, and physical activity. The mediation analysis was conducted using model 6 of Hayes PROCESS macro in SPSS (IBM Corp). Specifically, internet use served as the independent variable, physical activity level (ie, the MET score derived from the IPAQ) served as the dependent variable, and eHealth literacy and internet addiction were specified as mediators of the relationship between internet use and physical activity (Figure 1). In addition, age, sex, and BMI were included as control variables. Bootstrapping with 5000 resamples was performed [35]. The 95% CI was defined by the lower limit CI and upper limit CI. Mediation effects were considered statistically significant if the CI did not include zero. All statistical analyses were conducted using SPSS (version 29.0.2), and a *P* value of<.05 was considered statistically significant.

### Ethical Considerations

Ethics approval was obtained from the Institutional Review Board of The Hong Kong Polytechnic University (HSEARS20230701001). Before participation, parents were asked to provide electronic informed consent, and both students and their parents had to indicate agreement to participate. No compensation was provided to participants. All data collected were anonymized and stored in a password-encrypted database.

## Results

A total of 117 participants completed the survey. Of these, 61.5% (72/117) were girls. The mean age of the participants was 15.9 (SD 1.29) years. The majority (n=50, 42.7%) of the participants were secondary school year 5 students. On average, the participants reported spending 5.28 (SD 3.50) hours per day using the internet, and the mean eHealth literacy score was 31.15 (SD 4.04). Table 2 presents the characteristics and scores of the participants.

**Table 2.** Participant characteristics and scores of questionnaires (N=117).

	Values
Sex, n (%)	
Female	72 (61.5)
Male	45 (38.5)
Age (y), mean (SD)	15.9 (1.29)
BMI (kg/m^2^), mean (SD)	20.2 (3.05)
Form, n (%)	
1	7 (6)
2	2 (1.7)
3	11 (9.4)
4	35 (29.9)
5	50 (42.7)
6	11 (9.4)
Missing	1 (0.9)
Internet use per day (h), mean (SD)	5.28 (3.50)
Use technology to monitor health, n (%)	
Yes	36 (30.8)
No	81 (69.2)
Physical activity, mean (SD)	2667.65 (2657.73)
Internet addiction (possible range 26‐104), mean (SD)	63.00 (13.83)
eHealth literacy (possible range 8‐40), mean (SD)	31.15 (4.04)

Correlations between internet use, internet addiction, eHealth literacy, and physical activity are presented in Table 3. Internet use was significantly and positively correlated with internet addiction (*r*=0.33; *P*<.001) and negatively correlated with physical activity (*r*=−0.22; *P*=.02). eHealth literacy was negatively correlated with internet addiction (*r*=−0.26; *P*=.005) but was not correlated with internet use or physical activity. Internet addiction was negatively correlated with physical activity (*r*=−0.25; *P*=.006).

**Table 3.** Spearman ρ correlation between internet use, internet addiction, eHealth literacy, and physical activity.

	1	2	3	4
Internet use	—^a^	—	—	—
Internet addiction	0.33^b^	—	—	—
eHealth literacy	0.06	−0.12	—	—
Physical activity	−0.21^c^	−0.26^d^	−0.05	—

a Not applicable.

b *P*<.001.

c *P*<.05.

d *P*<.01.

**Figure 2.**
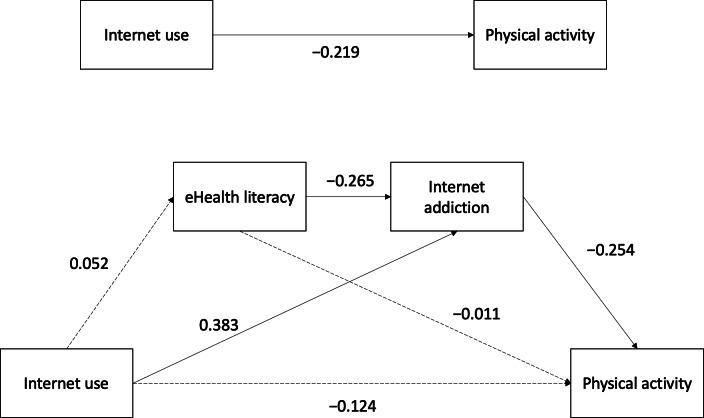
The mediation model’s results for internet use, eHealth literacy, internet addiction, and physical activity. All effect coefficients are reported using standardized coefficients. The solid line indicates a significant path; the dotted line indicates an insignificant path.

The results of the mediation analysis are presented in Table 4 and Figure 2. Internet use had a direct effect on internet addiction (*B*=1.53; *P*<.001). eHealth literacy also had a direct effect on internet addiction (*B*=−0.91; *P*=.002). Internet addiction had a direct effect on physical activity (*B*=−43.94; *P*=.02). The findings revealed neither a direct effect of internet use on physical activity or eHealth literacy nor a direct effect of eHealth literacy on physical activity. Only one significant indirect effect was observed: internet use was indirectly associated with physical activity through internet addiction (*B*=−67.19, 95% CI −148.17 to −9.45).

**Table 4.** Mediation analysis of the relationship between internet use on physical activity via eHealth literacy and internet addiction^a^.

	Unstandardized coefficients, *B* (SE)	*t* test (*df*)	*P* value	95% CI
Direct effect				
Internet use on physical activity	−85.81 (70.27)	−1.22 (105)	.23	−225.14 to 53.52
Internet use on eHealth literacy	0.052 (0.11)	0.46 (107)	.65	−0.17 to 0.27
Internet use on internet addiction	1.53 (0.35)	4.43 (106)	<.001	0.84 to 2.21
eHealth literacy on Internet addiction	−0.91 (0.29)	−3.12 (106)	.002	−1.50 to −0.33
eHealth literacy on physical activity	−6.47 (57.30)	−0.11 (105)	.91	−120.08 to 107.14
Internet addiction on physical activity	−43.94 (18.15)	−2.42 (105)	.02	−79.93 to −7.95
Indirect effect				
Internet use → eHealth literacy → physical activity	−0.3393 (6.74)	—^b^	—	−10.67 to 17.79
Internet use → internet addiction → physical activity	−67.19 (35.96)	—	—	−148.17 to −9.45
Internet use → eHealth literacy → internet addiction → physical activity	2.11 (5.36)	—	—	−10.05 to 13.16
Total effect				
Internet use on physical activity	−151.23 (65.76)	−2.30 (107)	.02	−281.58 to −20.88

a The model controlled for age, gender, and BMI. Analysis was based on 112 participants after listwise deletion of cases with missing data.

b Not applicable.

## Discussion

### Principal Findings

This study examined the relationships between internet use, eHealth literacy, internet addiction, and physical activity. In particular, we hypothesized that eHealth literacy and internet addiction mediate the relationship between internet use and physical activity. The results indicated that internet addiction was the only significant mediator in this relationship. Although our findings revealed that eHealth literacy was not associated with internet use and physical activity, it had a direct effect in reducing internet addiction.

Our results revealed no significant association between time spent on the internet and eHealth literacy. This finding suggests that spending more time online does not necessarily improve individuals’ ability to understand and use health-related information available online. This result contrasts with the findings of some previous studies. For instance, one study reported that more frequent use of information and communication technology was associated with a higher level of eHealth literacy [36]. Various factors may explain this discrepancy, including a lower level of trust in online health information than in information provided by clinicians [37] and the nature of online activities, which may be primarily entertainment oriented rather than health related [38]. In addition, experiences during the COVID-19 pandemic may have contributed to increased health awareness and, consequently, increased eHealth literacy [39]. Thus, the effect of recent internet use on eHealth literacy might be less pronounced than before. Overall, these findings indicate that although internet use is widespread, it does not automatically translate into improved eHealth literacy.

A significant negative relationship was observed between eHealth literacy and internet addiction, which supports one of our hypotheses. This finding indicates that a higher level of eHealth literacy is associated with a lower level of internet addiction. Individuals who are more familiar with searching for and understanding online health information might be more aware of the potential drawbacks of excessive internet use and thus may be better able to avoid behaviors that contribute to addiction [40]. This finding underscores the importance of enhancing eHealth literacy as a potential strategy for mitigating internet addiction and identifies a critical target for future intervention programs.

This study also examined the relationship between eHealth literacy and physical activity and found no significant relationship in either the correlation or the mediation analysis. Although previous studies have reported a positive relationship between eHealth literacy and physical activity [18,40], our results suggest that being literate in eHealth does not necessarily translate into increased engagement in healthy behaviors, such as physical activity. The lack of a significant relationship might be due to various external factors such as personal motivation, social influence, limited resources, academic demands, or other daily life commitments, which may play a more prominent role in affecting adolescents’ willingness and opportunities to engage in physical activity [41,42]. Therefore, although eHealth literacy is an important ability in terms of accessing and understanding health information, it does not appear to affect physical activity behaviors directly; it may thus reflect a gap between health-related intentions and actual behavior [43]. Moreover, the perceived need to engage in health-promoting behaviors might be another variable contributing to the nonsignificant results [44,45]. For instance, a study on the use of mobile health applications reported that individuals with severe obesity were more likely to use health applications to engage in health behaviors than individuals who were underweight [44]. In addition, a study by Weaver et al [45] examining the types of health information searching (wellness, illness, or mixed information) found that individuals who searched only for wellness-related information reported a better health status and a higher level of physical activity than those in other groups [45]. These findings indicate that although individuals may be confident in their ability to search for and use online health resources, not all individuals are motivated to change their lifestyle in favor of wellness. Some may consider themselves as healthy or may not prioritize health behavior changes at their current life stage. Individuals may possess eHealth literacy but engage with health information only when they perceive a need, such as searching for illness-related information when experiencing symptoms. The nonsignificant relationship between eHealth literacy and physical activity observed in this study might indicate that adolescents with eHealth literacy place less emphasis on promoting their wellness.

This study provides preliminary results regarding the use of health information from electronic sources among adolescents in Hong Kong. The level of eHealth literacy observed in our sample (mean 31.15, SD 4.04) was comparable to that reported in other countries, such as the mean score of 28.4 (SD 7.6) reported by Koo et al [29]. Although no official cutoff score exists for the eHEALS to define adequate eHealth literacy, a recent study on adults in China suggested a cutoff of 29.5 [46]. On the basis of this reference value, only 19.7% (23/117) of adolescents in this study scored below the suggested cutoff value, indicating that 80.3% (94/117) of our participants had adequate eHealth literacy. Furthermore, our results suggest that internet addiction could be reduced by increasing eHealth literacy. In addition to seeking health information online, adolescents also obtain health advice from school [47]. Thus, educators should consider incorporating electronic health information sources into classroom instruction or extracurricular activities. However, eHealth literacy was not associated with physical activity in this study. Future studies should examine the factors mediating the relationship between eHealth literacy and health-promoting behaviors. In particular, further investigation is needed to understand how eHealth literacy can be translated into actual health-related behaviors among adolescents.

This study has several limitations. First, all the measures were self-reported; therefore, the participants’ responses may have been influenced by social desirability bias or inaccurate due to recall bias regarding, for example, the time spent on the internet and physical activity. Second, this study included a relatively small sample from a few secondary schools in Hong Kong. Thus, its generalizability is limited. Future studies with larger and more diverse samples are recommended to increase representativeness among Hong Kong adolescents.

### Conclusions

This study investigated the relationships among internet use, eHealth literacy, internet addiction, and physical activity. The results provided preliminary evidence regarding the level of eHealth literacy among adolescents in Hong Kong. Furthermore, the results highlight the significant role of eHealth literacy in reducing internet addiction; however, eHealth literacy was not found to be associated with physical activity. Teachers and parents should promote adolescents’ eHealth literacy as a strategy to support health and prevent internet addiction.

## Abbreviations

CIAS: Chen Internet Addiction Scale
eHEALS: eHealth Literacy Scale
IPAQ: International Physical Activity Questionnaire
MET: metabolic equivalent of task
